# Hemodynamic effects of the combined support with VAV-ECMO, Impella CP, and Impella RP

**DOI:** 10.1007/s00392-023-02304-2

**Published:** 2023-09-20

**Authors:** Baravan Al-Kassou, Nils Theuerkauf, Georg Nickenig, Sebastian Zimmer

**Affiliations:** 1 Heart Center Bonn, Department of Medicine II, University Hospital Bonn, Venusberg-Campus 1, 53127 Bonn, Germany; 2 Department of Anesthesiology and Intensive Care Medicine, University Hospital Bonn, Bonn, Germany

Sirs:

Cardiogenic shock (CS) is a state of life-threatening end-organ hypoperfusion due to diminished cardiac output resulting from left, right, or biventricular heart failure [1, 2]. Recommended management strategies include fluid administration, vasopressors, and inotropes. However, these drugs increase myocardial oxygen consumption and afterload and are often ineffective [3]. In recent years, the use of temporary mechanical circulatory support (MCS) has increased in patients with CS. Options for acute circulatory support include the use of Impella devices to improve cardiac output as well as veno-arterial extracorporeal membrane oxygenation (VA-ECMO), supporting circulation and gas exchange [1, 4, 5]. These devices are frequently used in combination, to improve the hemodynamic support [6]. In the following, we report a case that demonstrates the complex pathophysiology of CS and the hemodynamic impact of different CMS, as well as the difficulty of their combined use.

A 73-year-old patient suffered cardiac arrest during analgosedation for surgical placement of a hemodialysis catheter in a community hospital. The clinical history of the patient included coronary artery disease, ischemic cardiomyopathy with a left ventricular (LV) ejection fraction of 29%, and a normal right ventricular function with a tricuspid annular plane systolic excursion (TAPSE) > 18 mm. Following initially successful cardiopulmonary resuscitation (CPR), the patient developed a refractory CS with circulatory failure, leading the treating physicians to ask for VA-ECMO-assisted transfer to our hospital. Upon arrival of our shock team, cannulation for the VA-ECMO was done under ongoing CPR. The patient was subsequently transferred to our hospital under VA-ECMO support.

The echocardiographic assessment after successful cardiac defibrillation showed no residual LV contractile function. Emergent coronary angiography revealed a distal in-stent thrombosis of the left anterior descending artery, requiring percutaneous transluminal angioplasty (Supplementary Videos 1 and 2). Additionally, a high-grade stenosis of the medial left circumflex artery was treated with a drug-eluting stent (Fig. 1A, Supplementary Videos 3 and 4). The patient had an aortic valve regurgitation resulting in constant retrograde blood flow into the LV under ECMO support (Supplementary Video 5). Intracardiac hemodynamic measurements indicated a significantly increased left ventricular end-diastolic pressure (LVEDP) of 65 mmHg. To unload the LV, a percutaneous transvalvular micro-axial flow pump (Impella CP^®^) was placed in the LV. With maximal support power, the LVEDP rapidly decreased. However, due to VA-shunting by the ECMO, filling of the LV was lower than the venting volume provided by the Impella, resulting in insufficient LV filling and blood pressure depression. Decreasing the Impella support power to P3 balanced in- and out-flow as shown in the LV pressure tracing (Fig. 1B). A pulmonary embolism was ruled out in the computed tomography (CT) angiography.

**Fig. 1:**
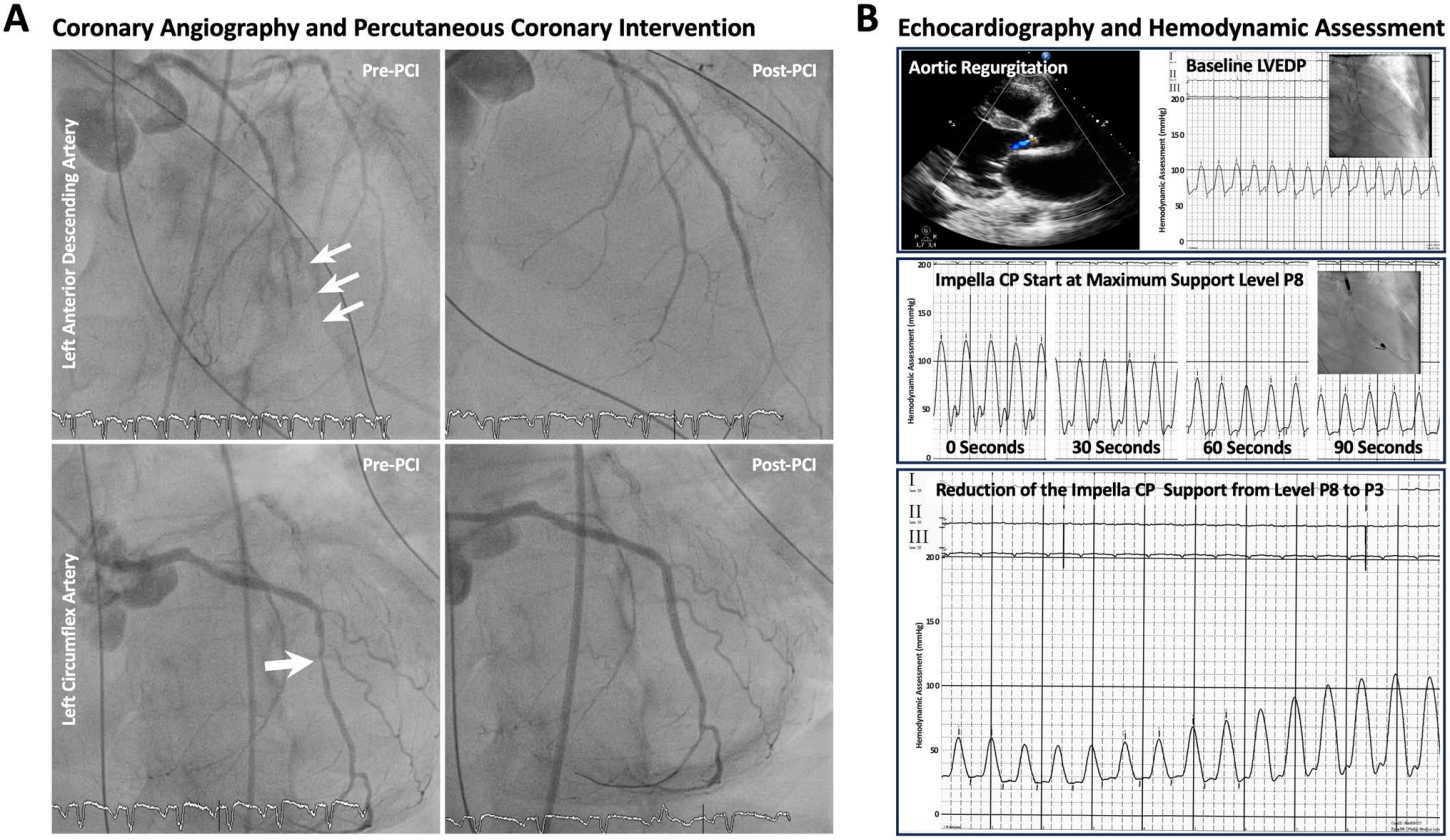
**A** Coronary angiography of the left coronary artery revealed an acute in-stent thrombosis in the distal left anterior descending artery and a high-grade stenosis of the left circumflex artery, that were treated by percutaneous transluminal angioplasty and drug-eluting stent implantation, respectively. **B** Intracardiac hemodynamic measurements showing improvement of the left ventricular end-diastolic pressure (LVEDP) using the Impella CP^®^ assist device

During the next 12 h, pulmonary ventilation decreased to < 3.0 ml/kg/min despite enhanced ventilation pressure gradients. Additionally, pulmonary blood flow decreased as detected in transesophageal echocardiography and via pulmonary artery catheter. The CT assessment showed a severe pulmonary deterioration with progressive consolidations of both lungs and limited pulmonary arterial enhancement, as compared to the previous day (Supplementary Video 6). Echocardiographic evaluation indicated a severe right ventricular (RV) failure with significant RV dilation. To unload the RV and enhance pulmonary perfusion, an additional right-sided axial flow pump (Impella RP^®^) was positioned in the pulmonary trunk. In Addition, a second outlet cannula was connected to the arterial line of the ECMO via Y-connector and placed in the right internal jugular vein (VAV-ECMO) to ensure oxygenated blood supply to the pulmonary circulation. Echocardiographic assessment showed an immediate cardiac improvement with significant reduction in RV dimensions achieved by increasing Impella RP^®^ flow (Fig. 2A). Furthermore, a rapid improvement in ventilation and gas exchange was observed, which was also reflected in the radiological X-ray images, showing a continuous regression of pulmonary consolidations (Fig. 2B).

**Fig. 2:**
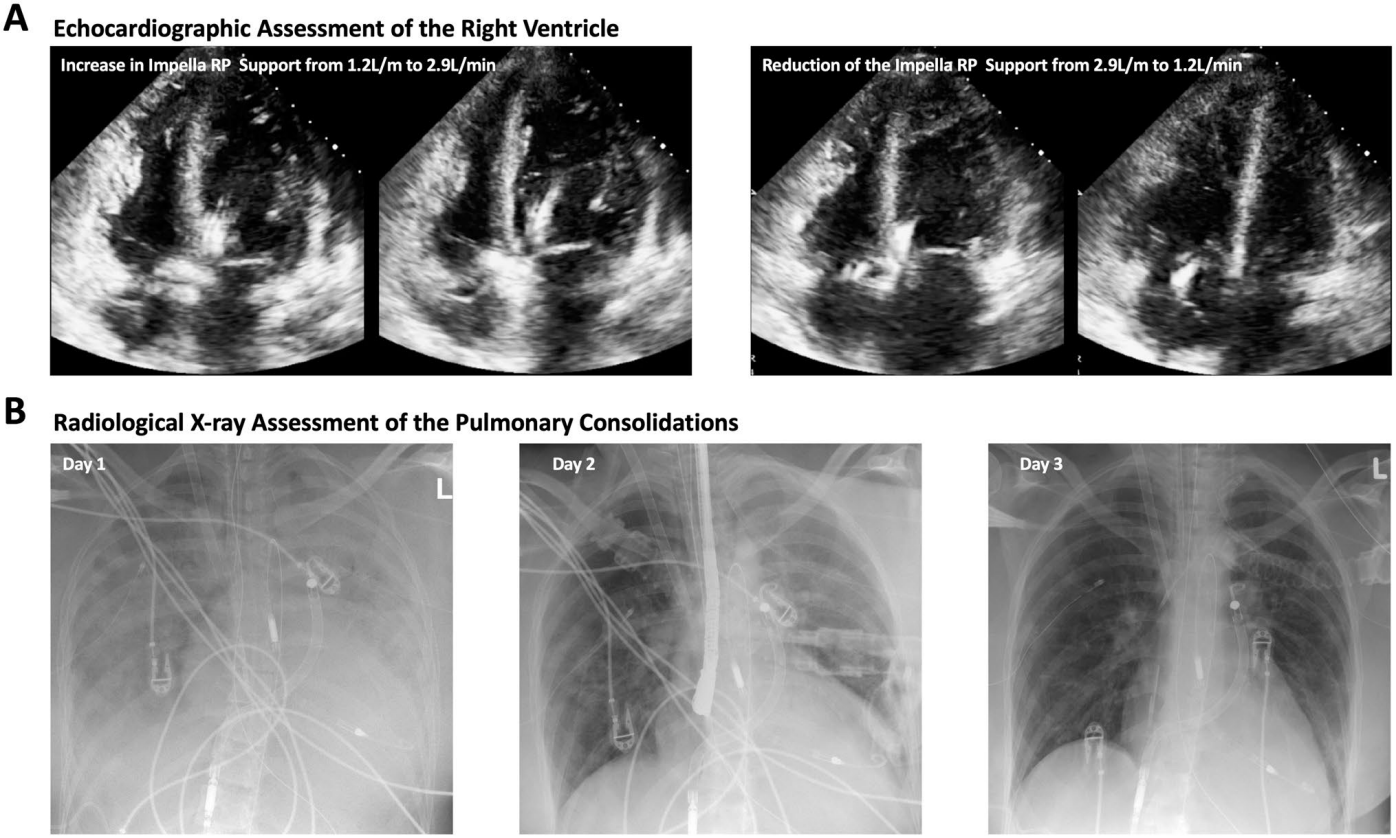
**A** Echocardiographic assessment showing immediate improvement as well as deterioration of the right ventricular function and dimensions depending on the Impella RP^®^ flow. **B** X-ray controls showing the initial pulmonary edema and consolidations on day 1, that significantly regressed over time using the Impella RP^®^ assist device

In the following days, the clinical situation improved, allowing for a reduction in inotropic therapy. On the sixth day after admission, the VAV-ECMO was safely removed. However, weaning from the axial flow pump was prolonged due to immediate RV dilation observed when reducing the Impella RP^®^ flow. Over the next six days, the flow rates of the axial flow pumps were gradually decreased as both left, and right ventricular function consistently improved. Eventually, the right-sided Impella RP^®^ could be successfully removed. Another four days later, the LV Impella CP^®^ was also removed (Supplementary Fig. 1). Echocardiographic assessment revealed a moderately reduced LV ejection fraction of 40% and a normal RV function, with a TAPSE > 18 mm.

Unfortunately, on the day of planed discharge, the patient developed refractory ventricular fibrillation. Emergent coronary angiography revealed adequate blood flow in the distal coronary arteries. Despite additional high-dose inotropic therapy and further defibrillations, a return of spontaneous circulation could not be achieved. In view of the patient's overall situation with a prolonged and severe course of the disease, the decision was made to terminate the CPR based on interdisciplinary consensus.

Cardiogenic shock is a multifactorial syndrome with high mortality [7]. The management of CS requires differentiated and individually adjusted therapy [1, 3]. Although proven to be effective in providing hemodynamic support, the use of VA-ECMO leads to a non-physiological circulation, sometimes resulting in increased LVEDP and LV dilatation [4, 8]. Consistently, in the present case, intracardiac hemodynamic assessment after VA-ECMO showed severely increased LVEDP. The insertion of a LV Impella resulted in an effective LV unloading with significant LVEDP reduction [5, 6]. Nevertheless, the patient developed RV failure with severe pulmonary consolidations. The use of an Impella RP^®^ was effective in managing the right-sided heart failure. Consistent with reports on patients with left ventricular assist device, the additive right heart support accelerated RV recovery and ECMO weaning [9]. Overall, although refractory CS remains the main indication for MCS, evidence demonstrating a clear benefit of this therapeutic approach is limited [10]. Randomized trials have not yet shown a survival advantage for patients with CS, regardless of whether ECMO or an Impella device was used [11, 12]. However, in the setting of ECMO-treated CS, Impella-supported LV unloading has been shown to decrease 30-day mortality [13]. In the present case, despite initial improvement with successful weaning from circulatory support, the patient unfortunately developed fatal cardiac arrest. Even in retrospect, preventive LVAD or ICD implantation would not have been the first choice, as LV function had improved to 40% and RV had completely recovered.

The use of temporary mechanical circulatory support for CS has evolved significantly. The present case report shows the complexity of CS and demonstrates the hemodynamic effects of different MCS, and the challenges arise when combining them. The combined use of VAV-ECMO, Impella RP, and CP was effective in managing the biventricular failure. However, the patient died after weaning from the circulatory support and significant clinical improvement, leaving us with the open question of potentially missed treatment measures.

### Supplementary Information

Below is the link to the electronic supplementary material.

Supplementary file1 Graphical Overview of the Patient’s Treatment Trajectory (TIFF 43651 KB)

Supplementary file2 (MOV 7088 KB)

Supplementary file3 (MOV 6778 KB)

Supplementary file4 (MOV 7977 KB)

Supplementary file5 (MOV 4115 KB)

Supplementary file6 (MOV 6244 KB)

Supplementary file7 (MOV 17155 KB)

## Data Availability

The data underlying this article will be shared on reasonable request to the corresponding author.
