# Supplementary figures and images for: Hemodynamic effects of the combined support with VAV-ECMO, Impella CP, and Impella RP
Source: Clin Res Cardiol. 2023 Sep 20;113(4):647–50. doi: 10.1007/s00392-023-02304-2 (PMC10954856; doi:10.1007/s00392-023-02304-2)

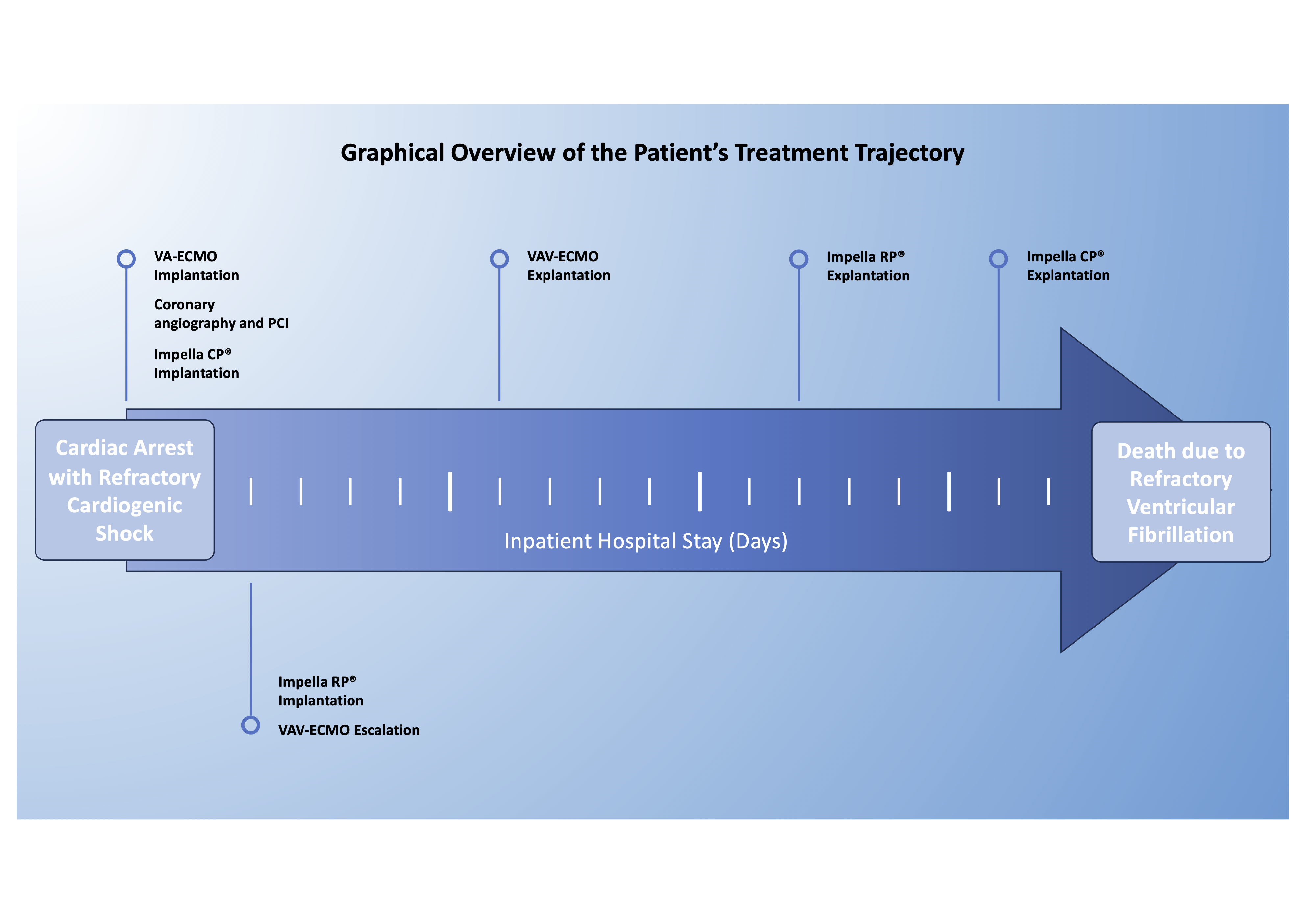

Supplement: Supplementary file 1 — Supplementary file1 Graphical Overview of the Patient’s Treatment Trajectory (TIFF 43651 KB) [file 392_2023_2304_MOESM1_ESM.tiff]
